# 

**Published:** 1878-05

**Authors:** 


					﻿Whole Number.
CCCXCVI.
May, 1878.
Number 5.
Vol. XXXVI.
THE
CHICAGO
MEDIC AL
1 - ' -
T ournal and Examiner
EDITORS:
WILLIAM H. BYFORD, A.M., M.D.
JAS. NEVINS HYDE, A.M., M.D. FERD. C. HOTZ, M.D.
E. FLETCHER INCALS, M.D.
CHICAGO:
PUBLISHED) BY THE CHICAGO MEDICAL PRESS ASSOCIATION.
188 JClark Street.
;f'Read the Notice of this Journal among Advertisements.
				

